# Giant Cell Arteritis and Cardiac Comorbidity

**DOI:** 10.7759/cureus.13391

**Published:** 2021-02-17

**Authors:** Magela Arias, Milad Heydari-Kamjani, Marc M Kesselman

**Affiliations:** 1 Osteopathic Medicine, Nova Southeastern University Dr. Kiran C. Patel College of Osteopathic Medicine, Davie, USA; 2 Rheumatology, Nova Southeastern University Dr. Kiran C. Patel College of Osteopathic Medicine, Davie, USA

**Keywords:** giant cell arteritis, cardiac comorbidity, large-vessel vasculitis and gca

## Abstract

Giant cell arteritis (GCA) is a large vessel vasculitis with a pathogenesis that involves two CD4 T-helper cell lineages, Th1 and Th17. The goal of GCA treatment is to achieve clinical remission and prevent complications, especially vision loss. Despite recent advances in treatment and diagnostic modalities for GCA, there continues to be a gap in the medical literature in addressing treatment and follow-up for patients with GCA after clinical remission is achieved. Of the most important issues to address in this patient population by rheumatologists and primary care physicians alike, is that of cardiovascular disease (CVD) risks in GCA patients associated with the vasculitis and its mainstay of treatment with high-dose glucocorticoids over a prolonged period of time. Physicians must be aware of the CVD events that have been observed in a higher proportion compared to the general population in GCA patients, including strokes, thoracic aortic aneurysms and dissections, myocardial infarctions, and peripheral vascular disease. This review will focus on the risk of CVD in GCA patients, with recommendations for management and follow-up.

## Introduction and background

Giant cell arteritis (GCA) is a type of granulomatous vasculitis, which predominantly affects the thoracic aorta and its major branches. The underlying mechanisms of disease activity involve both a Th1 and Th17-mediated damage to medium and large-sized arteries [1]. The classic presentation is that of a 50-year-old or older patient complaining of headache with new-onset vision changes including vision loss. Other presenting symptoms seen in GCA include jaw claudication, scalp tenderness, and fever [2]. The association of GCA with the feared complication of vision loss makes it one of the few rheumatologic emergencies in existence, requiring the need to properly diagnose and treat the condition [3]. In addition, the risk of cardiovascular morbidity and mortality is high in GCA as studies demonstrate that GCA patients are at a higher risk of mortality from cardiovascular disease (CVD) compared to the general population [4-6]. A higher rate of strokes, thoracic aortic aneurysms and dissections, ischemic heart disease, and peripheral vascular disease have been identified in patients with GCA [7,8]. Despite this association, limited data and research exist regarding management and follow-up options for CVD in GCA.

Diagnosis of GCA is commonly made using symptom assessment, medical history, laboratory testing, imaging, and temporal artery biopsy. Upon diagnosis, the goal of treatment is to prevent vision loss and achieve clinical remission. Typically, first-line treatment for GCA consists of high-dose glucocorticoids, of up to 60 mg of prednisone daily, with tapering until remission is achieved [9]. The use of high-dose glucocorticoids and the long treatment course is an added source of morbidity in these patients as steroids are associated with the development of osteoporosis, hyperlipidemia, diabetes, cataracts, infections, and CVD [10,11]. In recent years, the development of novel biologic therapy including the first Food and Drug Administration (FDA)-approved therapy specifically for GCA, tocilizumab, has provided patients with an option to achieve a steroid-sparing clinical remission. While some patients are able to achieve remission with glucocorticoids and biologics, many patients fail to achieve clinical remission, as evidenced by the Giant-Cell Arteritis Actemra (GiACTA) trial [12]. In addition, aspirin and statins have been evaluated as an adjunct therapy to glucocorticoids in GCA, but results have been mixed regarding their benefits in disease remission and mortality.

While the goal of treatment is to achieve and maintain clinical remission, there is limited data and guidelines on what to do for patients after they achieve remission, including when and whether steroids or biologics should be discontinued. In terms of follow-up, inflammatory biomarkers and symptoms have been historically used to evaluate disease flare ups and efficacy of treatment [13]. Meanwhile, imaging has shown evidence of a chronic inflammatory and vasculitic process in areas affected by GCA, despite patients experiencing clinical remission of symptoms and return of inflammatory biomarkers back to baseline after the use of either tocilizumab or glucocorticoids [13,14]. The long-term complications associated with the continued inflammation and vasculitis observed in imaging remain unclear. The advent of improved vascular imaging modalities are increasingly being utilized as alternatives to temporal artery biopsy for GCA diagnosis [15] and should be considered as a possible new means for monitoring clinically silent disease activity in patients under remission. Rheumatologists and primary care physicians (PCPs) caring for GCA patients need guidelines for follow-up and management for the potential risk of CVD events, especially in patients who are post-clinical remission. This review will focus on the risk of CVD in GCA patients, with recommendations for management and follow-up.

Epidemiology of giant cell arteritis and its implications in cardiovascular disease risks

A 2008 study estimated that 228,000 U.S. adults have been diagnosed with GCA [16]. All-cause mortality among GCA patients is similar to all-cause mortality within the general population. However, a meta-analysis demonstrated a statistically significant increased risk of mortality due to CVD in this patient population compared to the general population [17]. This association between mortality and CVD in GCA can be a result of the long duration of steroid treatment needed to achieve remission in these patients or due to lack of complete cessation of inflammation and vasculitis at the vessel walls under attack. In addition, cohort studies and meta-analyses have demonstrated increased risk of mortality and morbidity in GCA patients due to aortic dissections and aneurysms, cerebrovascular accidents, ischemic heart disease, and peripheral vascular disease compared to the general population [7]. Going forward, it is important for practicing clinicians, including rheumatologists and PCPs, to examine GCA patients for signs and symptoms of CVD in an effort to mitigate potential complications.

Pathophysiology of giant cell arteritis and its implications in cardiovascular disease risks

The underlying pathogenesis mechanism in GCA involves two CD4 T cell lineages, Th1 and Th17. Both lineages are mediated by different sets of interleukins (ILs) and cytokines. Th1 cell activation leads to increased levels of interferon gamma (IFN-gamma) and is implicated in the luminal stenosis of the vasculitic lesions and ischemic complications of the disease [18]. In addition, the involvement of Th1 cell lineage and IFN-gamma has also been cited in vascular inflammation and plaque formation in the settings of coronary artery disease [19]. Meanwhile, the Th17 response is mediated by IL-17 and promotes increased levels of IL-6. GCA is known for its high levels of acute-phase reactants, including C-reactive protein (CRP) and erythrocyte sedimentation rate. Research has found that IL-6 is the interleukin responsible for the increase in acute-phase reactants and is the target of the only biologic approved for GCA, tocilizumab. Evidence also has shown that the use of high-dose glucocorticoids in GCA patients significantly decreases levels of IL-6 (effectively decreasing levels of acute-phase reactants) but has no effect on IFN-gamma levels [1]. Although the Th1 mechanism remains activated in GCA patients during and after treatment, it remains unclear how the continued immune and inflammatory response observed affects patients over the long term. Meanwhile, there is an association between decreased levels of IL-6 and fewer systemic symptoms but increased incidence of vision and ischemic complications [1,20]. It can be inferred that when GCA patients present with ischemic issues and stenotic vessels, there is likely a greater Th1 response rather than a Th17 activation. This association highlights the dual pathway mechanism of the disease and the importance of developing a treatment regimen that tackles both Th1 and Th17 responses.

## Review

Cardiovascular disease risks associated with giant cell arteritis

Stroke

GCA patients have been shown to be at a 40% increased risk for strokes compared to the general population [21]. The risk of strokes in the general population is associated with the development of atherosclerosis and chronic inflammation. While GCA has not been found to accelerate atherosclerosis, the condition is associated with increased endothelial damage and ischemic complications due to the disease process and the treatment with glucocorticoids, resulting in increased risk for strokes in this patient population [22]. In particular, GCA patients are at a higher risk of vertebrobasilar strokes compared to the average individual who suffers from a stroke. Of clinical importance, there is a temporal association between GCA diagnosis and the type of stroke that patients suffer from. GCA patients tend to develop carotid territory strokes prior to or a month after the diagnosis. However, within the first month of diagnosis and treatment with glucocorticoids, there is an increased association with vertebrobasilar strokes [21]. Of patients with GCA who suffer vertebrobasilar strokes, the most predictive factors were a history of hypertension and irreversible vision loss [23]. Therefore, clinicians of patients who suffered from blindness due to GCA or have a prior history of hypertension apart from their GCA diagnosis should monitor those patients more closely for any signs of strokes. Additionally, further research is required on the effectiveness of preemptively treating these patients with preventative stroke medications such as antiplatelet therapy.

Thoracic Aortic Aneurysms and Dissections

GCA typically involves the thoracic aorta and its branches. This tropism for the thoracic aorta is further evidenced by the higher risk of thoracic aortic dissections and aneurysms observed in GCA patients compared to the general population [8]. Similar to stroke risk among GCA patients, a temporal association between the diagnosis of thoracic aortic dissections and aneurysms has been identified. The average time lapse between a diagnosis of GCA and thoracic aortic dissection was 1.1 years [24]. This is in contrast to the average of 10.9 years after the diagnosis of GCA when a thoracic aortic aneurysm, without rupture, is diagnosed [24]. A population-based study (n = 168) found that patients who developed thoracic aortic aneurysms and dissections when diagnosed with GCA had higher incidences of hyperlipidemia and coronary artery disease [25]. Of clinical importance, a murmur of aortic regurgitation was predictive of the diagnosis of thoracic aortic aneurysm in patients diagnosed with GCA [25]. The study by Nuenninghoff et al. (n = 168) found that among the 18 GCA patients diagnosed with thoracic aortic aneurysms and/or dissections, 10 had mild aortic regurgitation and seven had moderate aortic regurgitation [25]. There has been debate over whether and when GCA patients should be sent for imaging studies to evaluate whether they have aortic aneurysms, and the predictive value of listening to a murmur of aortic regurgitation should be known to clinicians as a screening tool for these patients.

Ischemic Heart Disease

Studies have provided conflicting information regarding the risk of coronary artery disease and acute coronary syndrome in GCA. Several studies found no statistically significant associations between coronary artery disease and GCA. This included a meta-analysis by Ungprasert et al. (n = 10,868) that combined several cohort studies and found no increased risk of coronary artery disease compared to matched controls [26]. Meanwhile, other studies showed the opposite correlation. This includes a cohort study by Tomasson et al. (n = 3,408) that found an elevated risk of myocardial infarctions, especially within the first month after the diagnosis of GCA [7]. Another population-based, cross-sectional study by Dagan et al. (n = 5,659) found an odds ratio of 1.247 of ischemic heart disease in GCA after taking into account other CVD risk factors, including diabetes mellitus, hypertension, hyperlipidemia, and smoking [27]. The risk of ischemic heart disease in GCA patients remains unclear and more research is warranted.

Peripheral Vascular Disease

In addition, a cohort study by Tomasson et al. (n = 3,408) found an increased risk of peripheral vascular disease in patients with GCA [7]. Specifically, data have demonstrated an increased association between peripheral arterial disease (PAD) and chronic inflammatory conditions as well as elevated CRP levels [28]. The risk for peripheral vascular disease was the highest at the six-month follow-up versus two-year follow-up after the diagnosis [7]. A meta-analysis by Ungprasert et al. (n = 9,789) found a pooled risk ratio of 1.88 for the development of PAD in GCA patients compared to the controls [28]. This elevated pooled risk ratio indicates that there is an increased risk for PAD in GCA patients. Therefore, signs and symptoms of PAD should be monitored in these patients to prevent PAD complications.

Giant cell arteritis treatment options and their implications in cardiovascular disease risk

Glucocorticoids

Despite advances in biologics, including FDA approval of tocilizumab for GCA, and a myriad of adverse effects tied to glucocorticoids, they remain first-line therapy for GCA [9]. Oftentimes, GCA patients require a long course of steroid treatment resulting in a higher risk for glucocorticoid-related adverse events. Glucocorticoids are associated with a higher risk for infections, osteoporosis, hyperlipidemia, diabetes mellitus, hypertension, and cardiovascular disease [11]. As mentioned earlier, steroids have been shown to inhibit the Th17 pathway in GCA but have minimal impact in the Th1 lineage, which results in chronically elevated levels of IFN-gamma [1]. Despite the ability of glucocorticoids to prevent blindness and provide clinical remission of GCA, their inability to suppress the Th1 pathway requires further investigation into the long-term outcomes of high IFN-gamma levels in GCA patients and whether it allows the continued progression of the illness.

Biologics

Currently, tocilizumab, an IL-6 alpha receptor inhibitor, is the only FDA-approved biological agent for GCA patients. It was approved based on the results of the GiACTA trial, a randomized controlled trial that found tocilizumab in combination with a 26-week tapering dose of prednisone demonstrated improved rates of clinical remission compared to either 26 or 52 weeks of prednisone taper plus placebo without the need to increase glucocorticoid dosing [12]. Most strikingly, the study showed that clinical remission rates were still quite low, especially for those on prednisone taper, which continues to be first-line treatment for GCA. The trial found that of those patients who were administered tocilizumab weekly, 56% achieved remission. This means that almost half of the participants had a flare of GCA, requiring further glucocorticoids and a longer treatment course. This is in comparison to the patients who were administered prednisone taper with a placebo of either 26 or 52 weeks, each of which resulted in a 14% and 18% achievement of clinical remission, respectively. Those involved in the GiACTA trial further evaluated the study characteristics and found that most patients who had a flare of the disease did so while still on glucocorticoid treatment, and that the addition of tocilizumab allowed for a faster control of the vasculitic process [29]. Even though tocilizumab allowed for clinical remission of the disease over the period of 52 weeks, imaging studies using magnetic resonance angiography revealed that the inflammatory process occurring at the vessels affected by GCA continued in approximately one-third of the patients [14]. They found that the clinically silent inflammatory process peaked closer to the initial treatment with tocilizumab and continued to decline [30]. This suggests that there is possibly a need to administer tocilizumab for a longer period of time to substantially reduce the immune and inflammatory response and maintain clinical remission. It is still not known for how long patients should be placed on tocilizumab. After the completion of the GiACTA trial, those patients continued to be monitored and some did indeed have flare ups of the disease after achieving remission at 52 weeks with tocilizumab [31]. While tocilizumab provides clinicians a new treatment modality to thwart the side effects of prolonged glucocorticoid treatment that might exacerbate CVD risks, further studies are warranted on appropriate dosing and duration of the biologic and whether subclinical disease that remains under the treatment with this agent is of any long-term consequence.

﻿Another biologic agent, ustekinumab, an IL-12 and IL-23 inhibitor, is being evaluated as a treatment option for GCA patients. Ustekinumab appears to be a promising biologic for these patients due to a mechanism of action that targets both arms of the pathogenesis in GCA. It has the ability to thwart both the Th1 and Th17 cell lineages responsible for the respective vessel damage and systemic symptoms observed in GCA. At this time, currently available data have provided conflicting results regarding its efficacy in GCA. In a 52-week prospective, open-label trial of 25 GCA patients who were administered ustekinumab after they had failed glucocorticoid treatment showed successful tapering of glucocorticoids at the end of the 52 weeks (six of the 25 patients were able to discontinue glucocorticoids completely) without any relapse [32]. Meanwhile, another prospective, open-label trial of 13 GCA patients with relapsing or newly diagnosed GCA was ended prior to its completion due to a high rate of clinical relapse and treatment failure [33]. Of the 13 patients who were enrolled in the study, seven failed tapering of the medication and most had relapsed by 23 weeks. Further research efforts should investigate why some participants failed to respond to ustekinumab.

Aspirin

﻿Aspirin is another medication that has been used in the management of patients with GCA, even though its effects have not been evaluated in any randomized controlled trial to date [34] and studies have demonstrated conflicting information in regards to the benefits of aspirin as an adjunct in the treatment regimen for GCA. Weyand et al. evaluated the effects of low-dose aspirin on biopsy-proven GCA tissue and found that aspirin was capable of reducing levels of IFN-gamma in vivo [35]. This suggests that aspirin not only can be used as an anti-platelet medication to prevent ischemic events in GCA but may potentially be used as an adjunct anti-inflammatory agent in these patients. Even with these findings of in vivo reduction of IFN-gamma in GCA, there have been no clinical trials to look at remission rates when aspirin is added as an adjunct therapy to either glucocorticoids or biologics. In addition, a retrospective study (n = 143) showed that ischemic events decreased in GCA patients taking aspirin without increased bleeding risk [4]. Meanwhile, other data demonstrate the contrary including a meta-analysis that involved six retrospective studies (n = 914). The meta-analysis indicated no increased benefit to lowering ischemic events in patients who took aspirin compared to those who were not taking the medication [36]. These conflicting results warrant further research in the use of aspirin as an adjunct medication for GCA treatment as well as its use to decrease CVD events in GCA patients.

Statins

Statins, β-hydroxy β-methylglutaryl (HMG)-CoA reductase inhibitors, are commonly used in the treatment of hyperlipidemia and prevention of CVD risk, especially among patients at high risk for stroke, myocardial infarction, and other CVD-related events. In addition, they are recommended for asymptomatic patients who have elevated CVD risks. High levels of CRP have been associated with increased risk of cardiovascular events, and statins are capable of reducing the level of this acute-phase reactant [37]. Studies have demonstrated that rosuvastatin, a high-intensity statin, is useful in reducing the number of cardiovascular events in asymptomatic patients without hyperlipidemia but with high levels of CRP [38]. This suggests that statins may be valuable in decreasing CRP levels in inflammatory diseases like GCA and can potentially play a role in preventing some of the cardiovascular events observed in these patients, especially when steroids and other anti-inflammatory medications are tapered off due to clinical remission. However, studies to date have reported mixed findings when it comes to the use of statins in GCA patients. A retrospective study by Narvaez et al. (n = 121) identified no benefit of statin use in patients with GCA including no difference in ischemic events or tapering of glucocorticoids among those patients on statin therapy and without it [39]. Two retrospective studies in France found some benefits in the use of statin therapy as an adjunct to glucocorticoids. One of the studies (n = 103) observed decreased hospitalizations due to cardiovascular events [40], while the other study (n = 103) indicated that statin therapy reduced the time to taper steroid treatment in patients with GCA [41]. The use of statins in GCA has also been debated due to the association of GCA with polymyalgia rheumatica and possible exacerbation of polymyalgia rheumatica with the use of statin therapy [42]. This implication should not be dismissed and clinicians who decide to place their patients with GCA on a statin should be aware of this potential interaction. Statin use in these patients should not be evaluated solely for its purpose as an adjunct therapy to glucocorticoids or biologics. It should further be investigated whether the use of statins post-clinical remission in patients with GCA decreases cardiovascular events in these patients and whether it allows a longer course of disease remission compared to having the patient without any therapy after eliminating steroids or biologics when clinical remission is achieved.

Recommendations

An estimated three million patients will be diagnosed with GCA in Europe, North America, and Oceania by 2050 due to an aging population, with GCA being a disease of the elderly [43]. This increased disease burden in the coming years suggests a dire need for further research into the short and long-term CVD risks in patients diagnosed with GCA including improved treatment and follow-up guidelines. Rheumatologists and PCPs managing GCA patients should be aware of the increased risk of CVD events, attributable to both disease pathophysiology and glucocorticoid treatment. The patient and clinician should be aware that these increased risks can arise, especially at the earlier stages when the disease is diagnosed due to the temporal association shown in previous studies regarding GCA diagnosis and CVD events. In terms of follow-up for GCA patients, clinicians should not only manage these patients to maintain clinical remission but should monitor patients and attempt to prevent CVD events, including strokes, thoracic aortic aneurysms and dissections, ischemic heart disease, and peripheral vascular disease.

**Figure 1:**
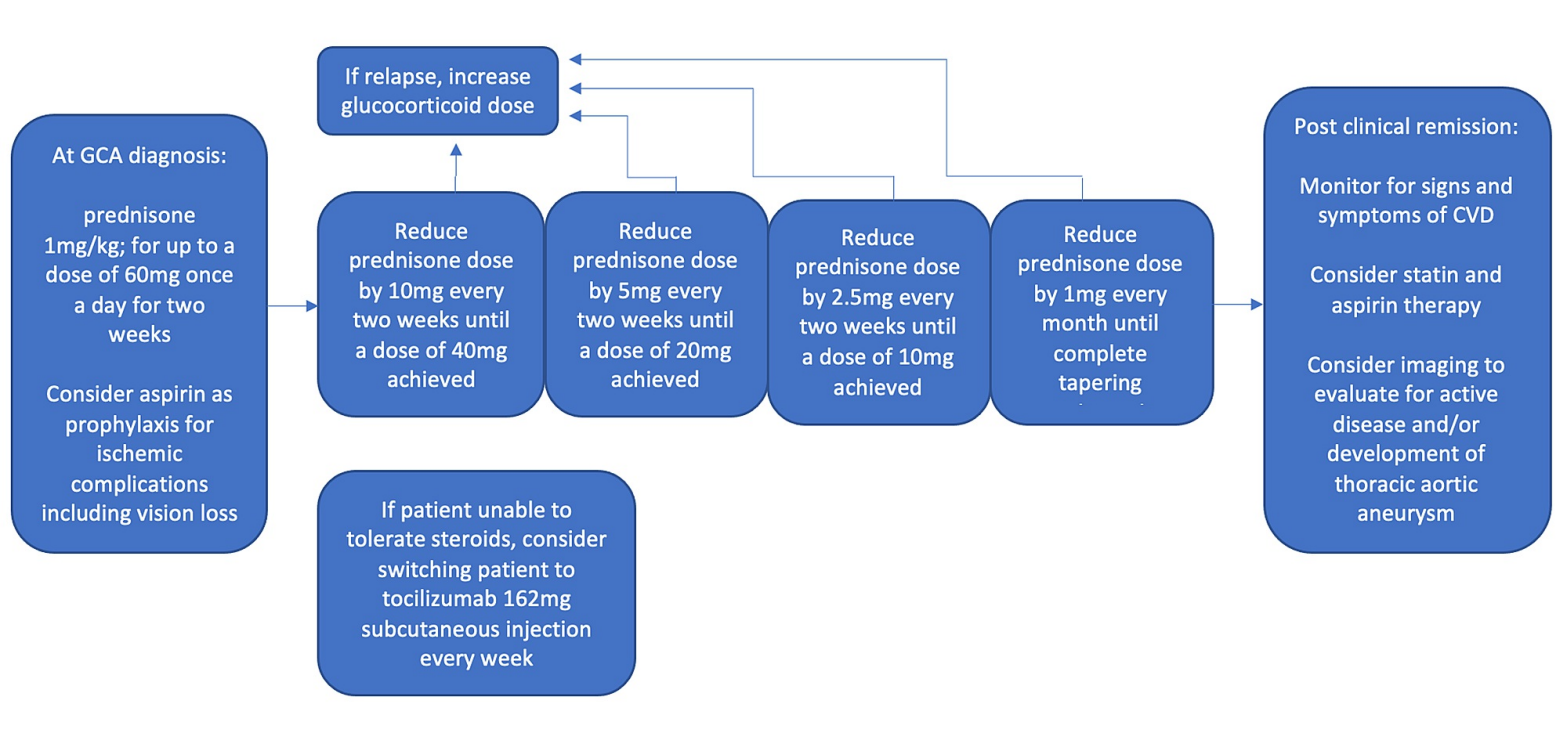
Recommended treatment and follow-up for GCA. GCA: giant cell arteritis; CVD: cardiovascular disease

In addition, GCA is a relapsing disease that can exacerbate after tapering off glucocorticoids or tocilizumab. Further studies are warranted to determine the significance of continued subclinical disease activity and its effects on long-term complications. Better indicators of disease exacerbation are also necessary. The use of biomarkers and clinical symptoms might not take into effect changes that are happening at the vessel wall level when patients are on either glucocorticoid or tocilizumab treatment. Improved imaging modalities have allowed the possibility to diagnose GCA without the need for a temporal artery biopsy [15]. It should be evaluated if there is a future role for imaging as another clinical tool to use to determine disease relapse. Figure 1 summarizes recommendations regarding treatment and follow-up in GCA.

## Conclusions

Future research for GCA should focus not only on steroid-sparing medications but also on medications that can be continued after remission is achieved to maintain patients in clinical remission for longer periods of time. Aspirin and statins have an established role in preventing CVD events and have anti-inflammatory effects that can be of benefit in the long-term treatment and management of GCA patients. Due to the lack of robust data on the use of these medications in GCA, further evaluation and research is required for their use as preventive CVD treatment in this patient population. This will allow for improved and ongoing GCA management with tailored medication regimens. To date, guidelines on how practicing clinicians should manage these patients once they achieve clinical remission remain sparse. Thus, organizations, clinical practitioners, and stakeholders should come together to develop more robust guidelines for the management of GCA patients upon achieving clinical remission that address how to mitigate CVD risks in GCA patients.
